# The Effect of Eating Sea Cucumber Jelly on *Candida* Load in the Oral Cavity of Elderly Individuals in a Nursing Home

**DOI:** 10.3390/md11124993

**Published:** 2013-12-11

**Authors:** Akira Yano, Akiko Abe, Fumie Aizawa, Hidetoshi Yamada, Kentaro Minami, Miki Matsui, Mitsuo Kishi

**Affiliations:** 1 Iwate Biotechnology Research Center, Kitakami, Iwate 024-0003, Japan; E-Mail: hyamada@ibrc.or.jp; 2 Division of Oral Health, Iwate Medical University School of Dentistry, Morioka 020-8505, Japan; E-Mails: aabe@iwate-med.ac.jp (A.A.); faizawa@iwate-med.ac.jp (F.A.); kminami@iwate-med.ac.jp (K.M.); matsuim@iwate-med.ac.jp (M.M.); mkishi@iwate-med.ac.jp (M.K.)

**Keywords:** *Stichopus japonicus*, holotoxin, *Candida*, oral care, elderly in nursing home

## Abstract

We conducted a double-blind randomized controlled study of elderly individuals in a nursing home to investigate the effect of the consumption of jelly containing sea cucumber on their oral *Candida* load. The jelly contained a hydrolysate of the sea cucumber *Stichopus japonicus*, which contained triterpene glycosides called holotoxins. The holotoxins worked as a fungicide, and their minimum inhibitory concentrations for *Candida albicans* were 7 µg/mL. Eight individuals in the nursing home took the sea cucumber jelly for a week and their oral *Candida* were counted before and after the intervention. Nine individuals took a control jelly without *S. japonicus*. The sea cucumber jelly showed inhibitory effects on the oral *Candida*. Thus, daily consumption of the *S. japonicus* jelly has the potential to reduce the oral *Candida* load in the elderly in nursing homes.

## 1. Introduction

*Candida* species are indigenous microorganisms present asymptomatically in a variety of different animal species. They can also cause severe infections in immunocompromised hosts [1]. *Candida* is the fourth leading microorganism responsible for human bloodstream infection in the USA and causes severe systemic candidiasis (deep mycosis), with 40% mortality [2]. *Candida* species initially colonize mucosal surfaces in the oral cavity, intestinal epithelia, or vagina, where they proliferate and cause mucosal infections in immunocompromised patients. Superficial candidiasis in the oral and oesophageal mucosa occurs in patients with HIV infection [3], transplant patients [4], and patients receiving cancer therapy, due to the increased susceptibility of these patients to fungal colonization and infection in upper gastrointestinal tract mucosa [5]. Some sequelae of oral candidiasis, including but not limited to ulcers, severe stomatitis, and mucosal hypersensitivity, reduce patient quality of life [6]. Furthermore, other physiological changes in the host can also lead to oral candidiasis. For example, the elderly are at increased risk of infection with opportunistic pathogens because of the higher incidence of several predisposing factors, such as systemic diseases, immunosuppression, use of medicines, use of dentures, and xerostomia, *etc.* [7,8,9]. *Candida* is more common in elderly individuals who require daily nursing care [10,11]. In addition, elderly individuals harboring *Candida* species orally are more likely to have oral mucosal lesions than those without *Candida* colonization [12]. According to the 2010 Japanese census, the elderly (65 or older) comprise 23% of the population. Furthermore, in Japan in 2009, 4,696,000 elderly individuals required long-term nursing care or living support [13]. Hence, control of *Candida* is a major concern in Japan, and other industrialized countries, due to the large elderly population.

Raw sea cucumber, *Stichopus japonicus*, has been eaten in Japan for many years [14]. Sea cucumbers are considered as a rich source of bioactive compounds and their use has been proposed in functional foods [15]. Shimada identified holotoxins as antifungal agents in *S. japonicus* in 1969 [16]. Holotoxins are triterpene glycosides that have strong membranolytic action against fungal membranes containing Δ^5^-sterols due to the formation of ion channels [17]. In this study, we tested the effectiveness of *S. japonicus* containing jelly as an antifungal food in a double-blind randomized controlled trial in elderly persons requiring long-term nursing in a nursing home.

## 2. Results

### 2.1. Antifungal Activities of *S. japonicus* Holotoxins

The minimum inhibitory concentrations (MICs) of the sample of holotoxin (HTX) extracted from *S. japonicus* from northern Japan against various *Candida* species were determined at concentrations that led to complete growth inhibition at 24 h (Table 1).

**Table 1:** Minimum inhibitory concentrations (MIC) of holotoxin (HTX) for several species of *Candida.*

Strain	MIC (µg/mL)
*Candida albicans* SC5314	7.0 ± 2.0
*C. albicans* JCM1542	7.0 ± 2.0
*C. tropicalis* JCM1541	14.0 ± 4.0
*C. glabrata* JCM3761	7.0 ± 2.0
*C. parapsilosis* JCM1612	8.0 ± 0.0
*C. krusei* JCM1609	16.0 ± 0.0

Table data show mean ± standard deviation (*n* = 4).

**Figure 1:**
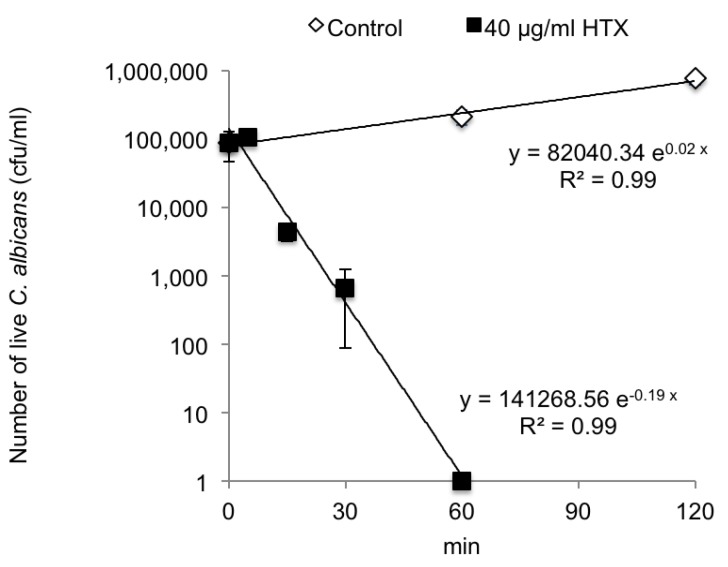
Time-killing curve of holotoxin for *Candida albicans* JCM1542. HTX at 40 µg/mL (black box) or dimethyl sulfoxide control (white box) were added to a log-phase culture of *C. albicans*. The number of viable cells at various time points was plotted (±standard deviation, duplicate analysis, repeated three times). Approximate curves and their formulae are also indicated, with coefficient values (*r*^2^).

To confirm the effects of HTX, a time-kill assay was performed on *C. albicans* JCM1542 (Figure 1). HTX was added at 40 µg/mL to the log-phase culture of *C. albicans*. The number of viable cells decreased exponentially until reaching the detection limit at 60 min. Therefore, this was a fungicidal, not fungistatic, time-kill curve.

We also tested the effects of HTX on several clinical isolates. Typically, two colony types were observed in the oral samples from the elderly individuals. The two colonies were identified as *C. albicans* and *C. glabrata* by TaqMan probe assay [18,19], and their susceptibilities to HTX were assessed by determining their MICs (Table 2). The MICs of the clinical isolates were similar to those of the laboratory strains.

**Table 2:** MIC of HTX for clinical isolates.

Strain	MIC (µg/mL)
*Candida albicans*	
SI_1	4.0 ± 0.0
SI_2	4.0 ± 0.0
SI_3	4.0 ± 0.0
SI_4	4.0 ± 0.0
SI_5	2.7 ± 2.3
SI_6	5.7 ± 4.0
*Candida glabrata*	
SI_11	10.7 ± 4.6
SI_12	8.0 ± 0.0
SI_13	10.7 ± 4.6
SI_14	10.7 ± 4.6
SI_15	13.3 ± 4.6
SI_16	8.0 ± 0.0

Table data show mean ± standard deviation (*n* = 3).

**Figure 2:**
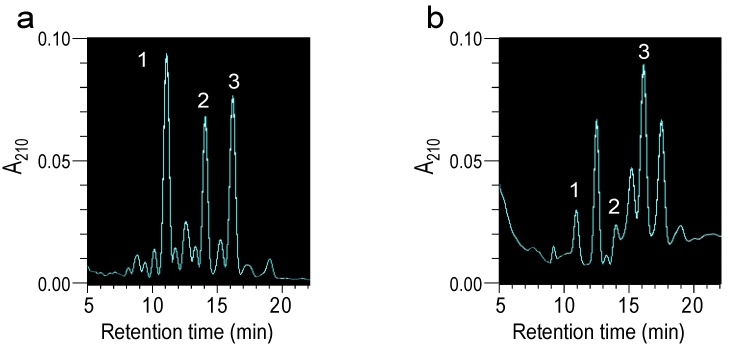
Analysis of HTX fractions and *S. japonicus* hydrolysate by HPLC and mass spectrometry. (**a**) Chromatogram of HTX. HTX was analysed by HPLC with an Inertsil ODS-3 column (4.6 × 150 mm) and a mobile phase with 35% acetonitrile at 0.8 mL/min. The major peaks were numbered from 1 to 3. (**b**) Chromatogram of the *S. japonicus* hydrolysate. The three peaks detected in HTX were also detected in the hydrolysate.

HTX contained three major peaks by HPLC (Figure 2a). The peaks were fractionated by HPLC, and antifungal activities were determined by micro-dilution assay (Table 3). Each fraction had similar antifungal activity to HTX. The LC-TOFMS analysis indicated that peak 1 contained the compound *m/z* = 1431.63, peak 2 contained *m/z* = 1445.65, and peak 3 contained *m/z* = 1415.64. The compounds in peaks 1 and 2 were detected as a Na^+^ ion and were deduced to be holotoxin B (C_66_H_104_O_32_; average mass = 1409.53) and holotoxin A (C_67_H_104_O_32_; average mass = 1423.55) [20]. Peak 3 was estimated as holotoxin A1 (C66H104O31; average mass = 1393.51) [21]. The mass spectra of each peak also contained the aglycone ion of holotoxin (C_30_H_44_O_4_).

**Table 3:** Antifungal activities of the HTX fractions and LC-TOFMS analysis.

HTX Fraction ^a^	MIC of Each Fraction (µg/mL) ^b^	A Compound Detected in Each Peak (Molecular Mass) ^c^
Containing peak 1	4.0 ± 0.0	1431.63
Containing peak 2	4.0 ± 0.0	1445.65
Containing peak 3	2.7 ± 1.2	1415.64

^a^ HTX was fractionated by HPLC with a CAPCELLPAK C-18 column (20 × 150 mm). ^b^ The fractions containing each peak were assayed by micro-dilution test with *C. albicans* JCM1542. The assay was done in duplicate and repeated three times. ^c^ HTX fractions were analysed by LC-TOFMS. Conditions for HPLC separation: InertSustain C-18 column (4.6 × 150 mm) and 0.8 mL/min with 35% acetonitrile. Ionization conditions: capillary voltage 3.1 kV, cone voltage 50 V, source temperature 120 °C, and desolvation temperature 300 °C. Dry nitrogen was used as the ESI gas.

Shimada reported that holotoxin had no antibacterial activity [16], and we also tested its effects on a variety of oral bacteria. The concentration of HTX was increased to 256 µg/mL, but this did not affect the growth of any of the tested bacteria: *Streptococcus oralis*, *Streptococcus sanguinis*, *Streptococcus gordonii*, *Streptococcus mutans*, *Actinomyces viscosus*, *Fusobacterium nucleatum*, and *Escherichia coli* (Supplementary Table S1). The bacteriostatic activity of HTX was also examined, but no effects were observed (Supplementary Figure S1).

### 2.2. Antifungal Activity of the Hydrolysate of *S. japonicus*

*S. japonicus* was hydrolysed to make a jelly for the clinical trial. The MIC of each lot of hydrolysate for *C. albicans* was determined, for which the values ranged from 3.13 to 50 mg/mL. Hydrolysates with MIC less than 6.25 mg/mL were used for the clinical trial. Comparisons of the MICs to antifungal drugs are shown in Figure 2a. The hydrolysates were analysed by HPLC and LC-TOFMS (Figure 2 and Table 4). The three HTX peaks were also detected in the hydrolysate (Figure 2b); however, the relative amounts of each peak were different from the *S. japonicus* samples. The hydrolysate in Figure 2b and Table 5, with a MIC for *C. albicans* of 6.25 mg/mL, was analysed by HPLC, and the amount of each peak was estimated by comparison with standard curves of each HTX fraction. The concentrations of peaks 1, 2 and 3 in the hydrolysate were 44.0, 51.0 and 263 μg/mL, respectively (Table 4). The three peaks in the hydrolysates contained compounds with the same molecular mass as in the HTX peaks, and were identified as holotoxin B, A and A1 by LC-TOFMS analysis (Table 4). The jelly contained 50% of hydrolysate, and the MIC was double that of the hydrolysate. Antifungal drugs had about 20-fold higher specific activities than HTX (Table 5).

**Table 4:** The deduced contents and molecular masses of the peak fractions in the hydrolysate.

	Deduced Concentration of Each Peak Fraction in the Hydrolysate (µg/mL) ^a^	A Compound Detected in the Peak of the Hydrolysate (Molecular Mass) ^b^
Peak 1	44.0	1431.63
Peak 2	51.0	1445.65
Peak 3	263	1415.64

^a^ The contents of the peak fractions in the hydrolysate (Figure 2b) were deduced by the HPLC chromatogram. ^b^ The hydrolysate was analyzed by LC-TOFMS. The LC-TOFMS conditions were the same as in Table 3.

**Table 5:** Antifungal activities of the hydrolysate and the jelly compared to antifungal drugs.

Sample	MIC (µg/mL)
The hydrolysate	6.25 ± 0.0 (×10^3^)
The jelly	12.5 ± 0.0 (×10^3^)
HTX	7.0 ± 2.0
Amphotericin B	0.4 ± 0.1
Miconazole	0.3 ± 0.2

### 2.3. Double-Blind Randomized Controlled Study

**Figure 3:**
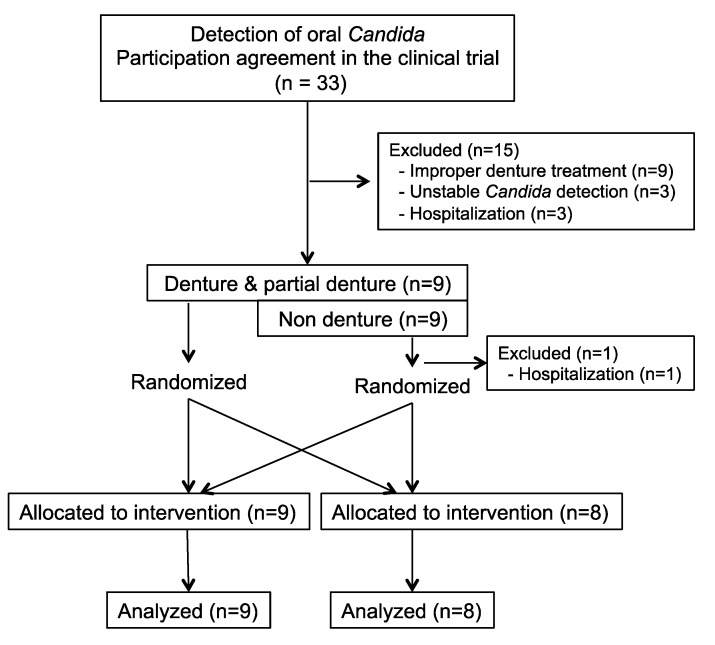
Flow diagram of the clinical study. The randomized controlled trial as designed to investigate the effect of sea cucumber jelly on oral *Candida*. We obtained participation agreements from 33 *Candida*-positive individuals. Oral *Candida* levels were measured before randomization. Three patients in whom oral *Candida* could not be detected were excluded. Three persons were hospitalized due to worsening physical health and did not participate in the study. Nine persons had problems managing their dentures (inability to clean their dentures, *etc.*) and were excluded. The remaining 18 persons were randomized to two groups. To maintain balance in the study population, the denture and non-denture groups were independently randomized. Before starting the intervention, one further participant was hospitalized and did not participate in the study. Two groups of participants (*n* = 9 and *n* = 8) underwent the control or *S. japonicus* interventions, and their oral *Candida* levels were compared.

The flow diagram of the clinical study is shown in Figure 3. Participation agreements were obtained from 33 candidates in whom *Candida* was detected in the oral cavity at examination 3 months earlier. Each candidate’s suitability for the trial was considered, and nine persons were excluded because they could not manage their dentures themselves. Three persons were hospitalized due to worsening physical health during the 3-month period. Another three persons were excluded because *Candida* were not detected when baseline colony values were counted. This left 18 participants for randomization, of whom nine used dentures and nine did not. Before randomization, one person was hospitalized due to worsening physical health.

**Figure 4:**
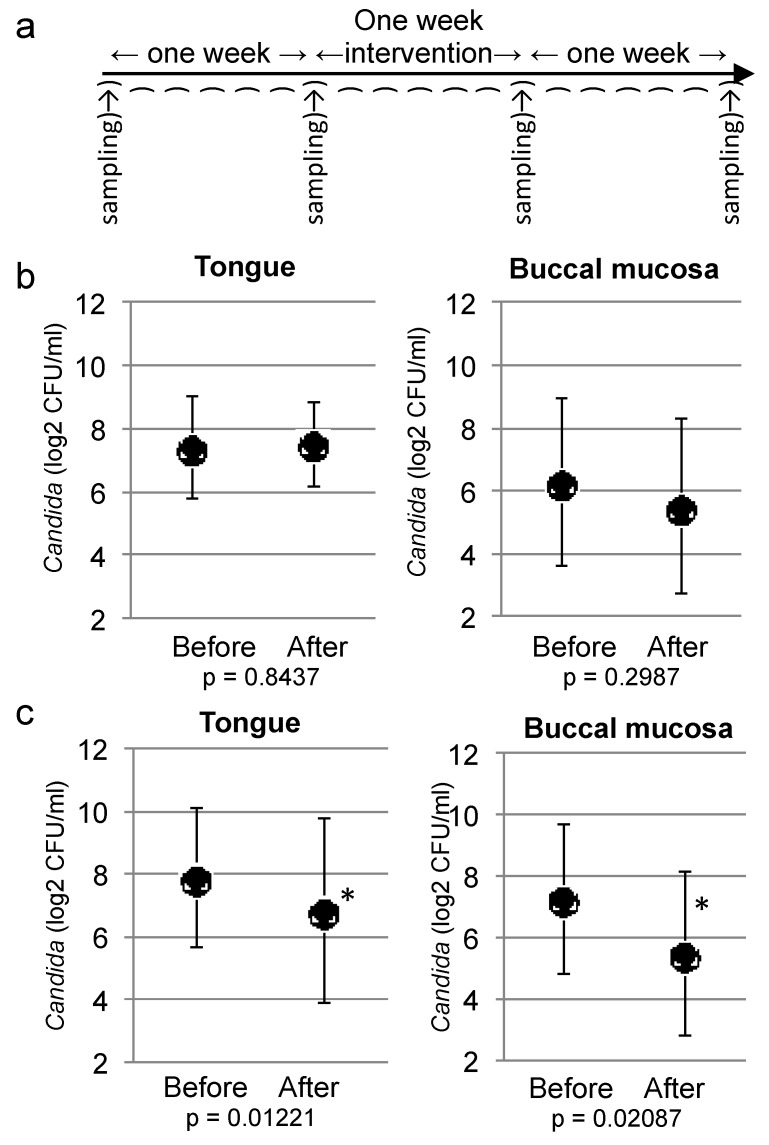
Effect of 1-week intake of *S. japonicus* jelly on *Candida* in the oral cavity of elderly individuals. (**a**) Schematic representation of the clinical trial. Swabs of the tongue and buccal mucosa were collected from subjects a total of 4 times: 1 week before starting jelly intake, several hours before starting jelly intake, 1 day after completion of jelly intake, and 1 week after completion of jelly intake. (**b**) *Candida* measured on the tongue surface and the buccal mucosa of the control group. The mean *Candida* CFU (log_2_ CFU/mL swab suspension) ± standard deviation (*n* = 9) is shown. The averages of the sampling before and after the control jelly intake are shown, with *p*-values from a paired *t*-test. (**c**) *Candida* measured on the tongue surface and the buccal mucosa in the test group. The mean *Candida* CFU (log_2_ CFU/mL swab suspension) ± standard deviation (*n* = 8) is shown. The averages of the sampling before and after the *S. japonicus* jelly intake are shown, with *p*-values from a paired *t*-test. * *p* < 0.05.

The baseline *Candida* colony value for each participant was determined by sampling 1 week before, and again about 2 h before the start of the intervention (Figure 4a). The elderly individuals were randomly assigned to the two groups, except that the number with dentures was split equally between the groups. The health status of the participants was generally poor, and they required nursing on a daily basis. Most participants took several medicines for control of blood pressure, blood glucose, lipids, depression, and dementia, and their oral mucosae were somewhat dried. All participants had been eating a soft-food diet. The nursing staff cleaned the oral cavity after each meal by tooth brushing. The numbers of *Candida* varied markedly between individuals, from 1 cfu/mL to over 1000 cfu/mL (uncountable). The number of colonies on the Sabouraud Dextrose Agar with antibiotics and the CHROMagar were similar, and the colony numbers on the CHROMagar were used for analysis. Typically, two colony types were observed on the CHROMagar plates: green colonies of *C. albicans* and pale brown colonies of *C. glabrata* [19]. Their identities were confirmed by TaqMan probe assay [18]. About 55% of the colonies were green. In five individuals (29% of plates), the plates contained only green colonies. Both types of colonies were counted as *Candida*.

After the second baseline sample was collected, the 1-week intervention was started. Twelve grams of jelly containing 25% (w/w) *S. japonicus* were provided for the test group after every meal as a dessert. Jelly without *S. japonicus* was provided for the control group. All participants were asked to eat the jelly without the denture. One day after finishing the intervention, a third *Candida* sampling was done, and a fourth sampling was done 1 week after completion of the intervention (Figure 4a).

The number of *Candida* colonies in the participants was statistically analyzed. The baseline was the average number of the first and second sampling, and the result was compared to the average of the third and fourth sampling. First, the normality of the distribution was assessed by Kolmogorov-Smirnov test (tongue of control group, *p* = 0.979; buccal of control group, *p* = 0.979; and tongue of test group, *p* = 0.660, buccal of test group, *p* = 0.087); all groups were estimated to have a normal distribution. The variance of the distribution was compared between test and control groups by *F* test (tongue of control group, *p* = 0.620; buccal of control group, *p* = 0.897; tongue of test group, *p* = 0.472; buccal of test group, *p* = 0.826); all groups were estimated to have the same distribution. The difference in colony number before and after the intervention was compared by paired *t*-test. *Candida* colony numbers were not different after the control intervention (Figure 4b). By contrast, *Candida* on the tongue and buccal mucosa were significantly decreased after 1 week of eating *S. japonicus* jelly (tongue, *p* = 0.01221; and buccal, *p* = 0.02087; Figure 4c).

## 3. Discussion

Food, especially in jelly form, can easily be taken by individuals without diagnosis or prescription by medical or dental doctors; thus, the prophylactic effect of *S. japonicus* jelly could benefit many elderly individuals and others at risk of *Candida* infection. Conventional mouthwashes that target oral microbes, including fungi, are another choice for prophylactic oral care. However, some chemical components of mouthwashes have a stimulating component that is uncomfortable for elderly individuals. Indeed, ethanol-containing rinses are not recommended for cancer patients in the oral mucositis guidelines [22,23]. Thus, this functional food based on *S. japonicus* has significant advantages over clinical drugs or mouthwashes if it can control fungi without inhibiting growth of resident microbiota or causing damage to mucosal tissues.

Thrice-daily consumption of the *S. japonicus* jelly reduced the average number of *Candida* to one-third of the pre-treatment number on the tongue and one-quarter on the buccal mucosa. The differences were statistically significant; however, the number of participants was limited and larger studies are required to confirm that sea cucumber jelly can reduce oral *Candida* in elderly patients. The reduction in the number of *Candida* was not as drastic as could be achieved with antifungal drugs, which would be expected to cure oral candidiasis within 1 week [24]. The jelly would also be effective in healthy people: 2 weeks of jelly intake reduced *Candida* to undetectable levels in healthy volunteers in our preliminary test (Supplementary Table S2). During our study, several persons were excluded before the intervention because of hospitalization due to worsening physical health. The physical condition of the elderly in the nursing home was often unstable, which could potentially affect the oral *Candida* load. To obtain clear results, increasing the number of participants and selection of more healthy *Candida*-positive persons will be necessary. In the case of our study, if the nursing staff had been able to manage the dentures of all participants, we could have increased the number of participants. However, the dentures of several individuals were apparently colonized by *Candida*, and decolonization of *Candida* in dentures usually requires strict oral management [25].

The jelly contained *S. japonicus* hydrolysate as an active food ingredient without concentration of any specific compounds and can therefore be classed as a processed or functional food, not a medicine. This is a major advantage in bringing this product into clinical use since the regulatory barriers are much lower for functional foods than for medicines.

The holotoxins were fungicidal, not fungistatic, as shown in the time-kill curve [26,27]. Holotoxins have no reported bactericidal activity. To confirm the lack of effect of holotoxins on oral bacteria, MICs were determined and effects on bacterial growth were tested. The results suggested that eating *S. japonicus* might specifically reduce oral *Candida*. We also confirmed that bacteria could grow in the hydrolysate (data not shown). The results suggested that eating *S. japonicus* might specifically reduce oral *Candida* without having any detrimental effect on the normal oral bacterial flora.

The daily amount of *S. japonicus* consumed by each subject in our study was estimated to be only about 18 g (fresh weight). Considerably more *S. japonicus* (estimated to over 100 g fresh weight of each sea cucumber) is commonly eaten in one serving in Japan or China, and there is no evidence that long-term consumption of *S. japonicus* has any deleterious effects [14]. Collagen is a major edible component of *S. japonicus* [28], and its hydrolysate, mainly containing collagen peptide, would be effective in preventing the oral mucosa from drying out [29], thus protecting a weakened mucosa. The oral mucosa of the elderly in nursing homes is usually dry due to a reduction in saliva production. We did not investigate the effect of *S. japonicus* on mucosal dryness in the oral cavity in this study, but it should be investigated in future trials.

## 4. Experimental Section

### 4.1. Fungal Strains

*Candida albicans* SC5314 was obtained from American Type Culture Collection (ATCC). *C. albicans* JCM1542, *C. tropicalis* JCM1541, *C. glabrata* JCM3761 and *C. parapsilosis* JCM1612 were obtained from Japan Collection of Microorganisms (JCM, RIKEN, Tsukuba, Japan). RPMI media was purchased from Lonza Japan (Tokyo, Japan). Other media and chemicals used in this study were purchased from Becton Dickinson (Franklin Lakes, NJ, USA), Sigma-Aldrich (St. Louis, MO, USA) and Wako Pure Chemicals (Osaka, Japan).

### 4.2. Antifungal Activities of Holotoxin

The holotoxin from *S. japonicus* was obtained from Holothurin Pharmaceutical Inc. (Osaka, Japan). The purity of holotoxins in the HTX was estimated to be up to 90% (w/w). Susceptibility testing of *Candida* was performed in accordance with Clinical and Laboratory Standards Institute-recommended procedures [30]. *C. albicans* JCM1542, *C. albicans* SC5314, *C. tropicalis* JCM1541, *C. glabrata* JCM3761, and *C. parapsilosis* JCM1612 were inoculated in RPMI and a micro-dilution assay with 24 h complete inhibition was used as an MIC index.

Time-killing activity of HTX for *C. albicans* JCM1542 was determined at higher concentration than the MIC [26,27]. DMSO (2% v/v) control or 40 µg/mL HTX were added to log-phase *C. albicans* cultures, with sampling at 5, 10, 30, 60, 90 and 120 min. Cultures were serially diluted 100-fold in sterile saline. Fifty microliter aliquots were subsequently plated on YPD agar plates. Colony counts were determined after incubation of the plates at 35 °C for 48 h. The assay was performed in duplicate and repeated at least three times.

### 4.3. Preparation and Analysis of *S. japonicus* Hydrolysate

*S. japonicus* obtained from the northern area of Japan (Iwate and Hokkaido prefectures) in the winter season of 2012 were purchased from Kawashu Ltd. (Iwate, Japan). Their internal organs were removed and a hydrolysate of the whole bodies was prepared by incubation with an equal weight of water with 0.5% (v/v) protease (Protease M Amano SD, Amano Enzyme Inc., Nagoya, Japan). The hydrolysate was sterilized by boiling and stored at −20 °C. The MIC of the hydrolysate for *C. albicans* was determined by micro-dilution assay as described above. Pre-treatment of the hydrolysate for HPLC and mass spectrometry was performed as follows. The hydrolysate was diluted 10-fold in water, applied to an HP20 (DIAION, Mitsubishi-Chemical, Tokyo, Japan) open column, and washed with 50% methanol. Holotoxins were eluted with methanol. The HPLC was performed using an Inertsil ODS-3 column (4.6 × 150 mm; GL Sciences, Tokyo, Japan) with a mobile phase containing 35% acetonitrile and monitoring of UV spectra at 210 nm. The flow rate was 0.8 mL/min and the column temperature was 35 °C. Three major peaks of HTX were fractionated by CAPCELLPAK C-18 (20 × 150 mm; Shiseido, Co., Ltd., Tokyo, Japan) with 15 mL/min 35% acetonitrile and used as standards.

Time of flight mass spectrometry (LC-TOFMS; Agilent Technologies, Palo Alto, CA, USA) was performed using a modification of the methods by Dyck *et al.* [31,32,33]. Briefly, liquid chromatography was performed using an InertSustain C-18 column (4.6 × 250 mm; GL Science Inc., Tokyo, Japan) with gradient elution using 0.1% formic acid and acetonitrile (20 to 50% of acetonitrile in 15 min) at a flow rate of 0.2 mL/min. Compounds were identified using Agilent Mass Hunter Workstation Software (Agilent Technologies Inc., Santa Clara, CA, USA).

### 4.4. *S. japonicus* Jelly

The MICs of the hydrolysates to *C. albicans* were determined as described above. Hydrolysate with a MIC lower than 0.5% (v/v) was used for making jelly. The jelly was made with 50% (w/w) of the hydrolysate, 5% aspartame (Pal Sweet, Ajinomoto Co., Inc., Tokyo, Japan), 10% gelatin (YASU CHEMICAL, Inc., Yasu, Japan) and 10% fruit juice (DOVER Ltd., Tokyo, Japan). The control jelly was made without the hydrolysate. The final amount of *S. japonicus* was 3 g (fresh weight) in one 12 g jelly (25%)*.*

### 4.5. Participants

The study was approved by the Ehics Committee of Iwate Medical University School of Dentistry (approval number D-01183). Informed consent forms were obtained for all 63 participants. Prior the intervention, it was determined whether or not *Candida* was present in the oral cavity of all subjects. The intervention group included 8 subjects (72–102 years old, median 87 years; 3 males, 5 females; 4 dentures, 6 dentate, 2 edentulous) and the placebo group included 9 subjects (78–96 years old, median 87 years; 1 males, 8 females; 4 dentures, 5 dentate, 4 edentulous). All participants required nursing for a range of reasons, such as cerebral infarction, cardiac failure, hypertension, hyperlipidaemia, cancer, dementia, rheumatoid arthritis, osteoporosis, cerebral contusion, and diabetes *etc.*, and were taking a number of medications. Also add inclusion and exclusion criteria for selecting study participants.

### 4.6. Sampling and Measurement of Oral Candida

Measurement of oral *Candida* was performed as described by Shinozaki *et al.* [19] with modifications for elderly requiring nursing. Both sides of the buccal mucosa were swabbed 5 times with a wet cotton tip, and the upper side of the tongue was also swabbed 10 times. Each cotton swab was immersed in 2 mL PBS and vortexed for 1 min; 0.5 mL of the suspension was then plated on CHROMagar Candida (Becton, Dickinson and Company, Franklin Lakes, NJ, USA) and Sabouraud Dextrose Agar with chloramphenicol and gentamicin (Becton, Dickinson and Company, Franklin Lakes, NJ, USA). Plates were incubated at 35 °C for 48 h, followed by counting of *Candida* colonies. Sampling of the participants started at 11 a.m. and finished before lunch. The team who have conducted the measurement of candida was blinded they did not know which participants received active products or placebo. The two typical colony types on the CHROMagar plate were isolated as clinical strains. The colonies were confirmed as *C. albicans* and *C. glabrata* by TaqMan probe assay [18].

### 4.7. Interventions

Participants were randomized to *S. japonicus*-containing jelly or placebo jelly groups and were asked to eat one jelly as dessert after every meal. Most of the participants were not able to feed themselves and received assistance from nursing staff, who did not know which type of jelly each individual received. Participants using dentures remove them when eating the jelly. The dentures were cleaned each day with an antifungal cleaner, Polident MicroClean (GlaxoSmithKline, Brentford, UK). The jelly intervention continued for 7 days (21 meals).

### 4.8. Statistical Analysis

Statistical analyses were conducted using R software [34]. The colony numbers of *Candida* were log-transformed prior to analysis. Before comparison of the test and control groups, the normality of the distribution was tested with the Kolmogorov-Smirnov test. The variance of the distribution between groups was compared using the *F* test. Finally, the *Candida* numbers before and after the intervention were compared using a paired *t*-test.

## 5. Conclusions

Thrice-daily eating of *S. japonicus* jelly reduced the number of oral *Candida* in elderly individuals in a nursing home. It was difficult to show the more clear effects of the jelly by the diversity of oral and medical condition of the subjects. To obtain conclusive results, clinical studies with larger sample size would be necessary.

## Supplementary Files

Supplementary File 1 Supplementary Information (PDF, 153 KB)
