# Supplementary material for: The Effect of Eating Sea Cucumber Jelly on Candida Load in the Oral Cavity of Elderly Individuals in a Nursing Home
Source: Mar Drugs. 2013 Dec 11;11(12):4993–5007. doi: 10.3390/md11124993 (PMC3877898; doi:10.3390/md11124993)

# Supplementary Information

**Table S1.** MIC of HTX for various bacteria.

**Table S2.** The effects of *S. japonicus* jelly on healthy volunteers**.**

**Figure S1.** Effects of HTX on bacterial growth. Growth curves of the Gram-positive bacterium *S. oralis* (**A**) and the Gram-negative bacterium *E. coli* (**B**), measured as $OD_{600}$ (±standard deviation, duplicate analysis, repeated three times), at several concentrations of HTX, as indicated in the graph.

**Table S1.** MIC of HTX for various bacteria.

| | **MIC (μg/mL)** |
|---|---|
| Gram-positive bacteria | |
| *Streptococcus oralis* ATCC10557 | >256 |
| *S. sanguinis* JCM5708 | >256 |
| *S. gordonii* DL1 | >256 |
| *S. mutans* UA159 | >256 |
| *Actinomyces viscosus* JCM8353 | >256 |
| Gram-negative bacteria | |
| *Fusobacterium nucleatum* JCM8532 | >256 |
| *Escherichia coli* DH5α | >256 |

**Table S2.** The effects of *S. japonicus* jelly on healthy volunteers.

| **Volunteer** | | **A** | **B** | **C** |
|---|---|---|---|---|
| *Candida* | Before intervention | 44 | 9 | 3 |
| (cfu/mL) | After intervention | 0 | 0 | 0 |

Three healthy volunteers (**A**, **B**, **C**) ate jelly containing *S. japonicus* for 2 weeks. The mouth wash solution (MW) was collected by gargling with 30 mL $H_2O$. MW was concentrated to 10 mL by collecting microbes by centrifugation. The number of oral *Candida* were indicated as number of colonies formed by 0.5 mL of MW on the CHROMagar Candida plate incubated at 35 ℃ for 48 h.

**Figure S1.** Effects of HTX on bacterial growth. Growth curves of the Gram-positive bacterium *S. oralis* (**A**) and the Gram-negative bacterium *E. coli* (**B**), measured as $OD_{600}$ (±standard deviation, duplicate analysis, repeated three times), at several concentrations of HTX, as indicated in the graph.

**A**

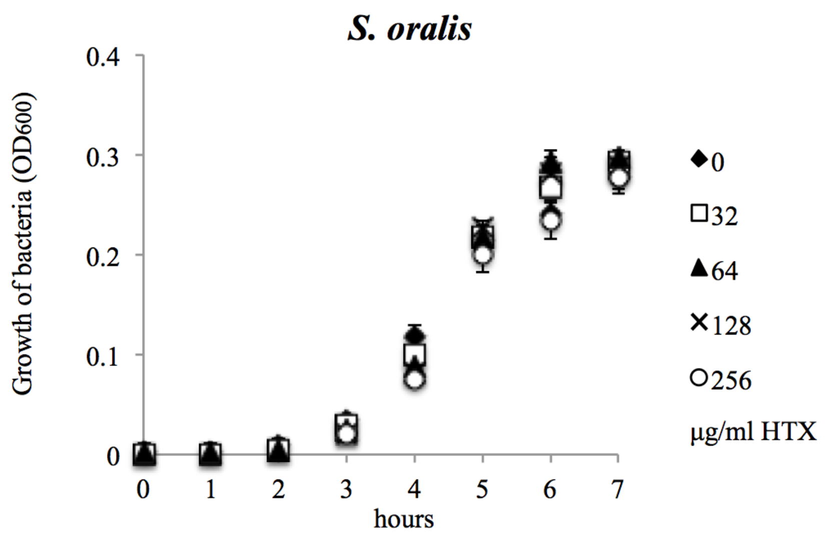


**B**

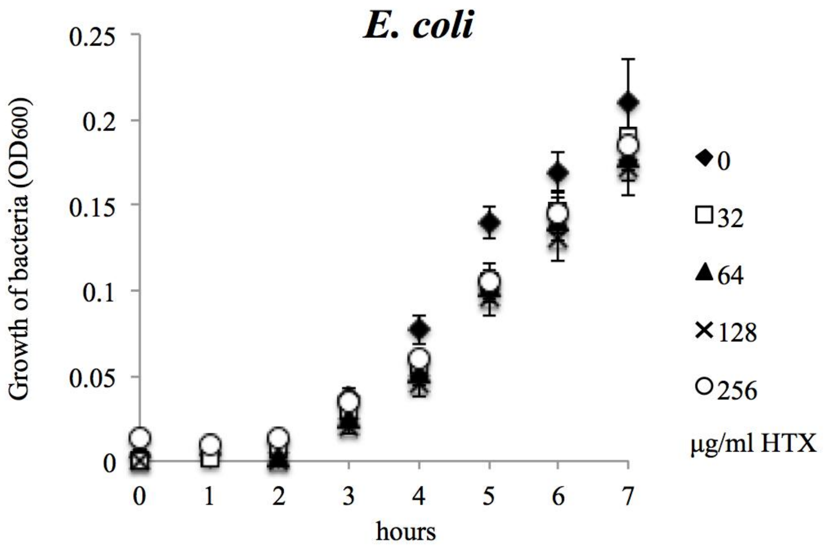

Supplement: Supplementary File 1 — Supplementary Information (PDF, 153 KB) [file marinedrugs-11-04993-s001.pdf]
